# T-PLL Presenting with an Indolent Course

**DOI:** 10.1155/2024/7310135

**Published:** 2024-07-24

**Authors:** Arsa Thammahong, Narittee Sukswai, Chantana Polprasert

**Affiliations:** ^1^ Department of Medicine Faculty of Medicine Chulalongkorn University and King Chulalongkorn Memorial Hospital, Bangkok, Thailand; ^2^ Center of Excellence in Antimicrobial Resistance and Stewardship Department of Microbiology Faculty of Medicine Chulalongkorn University, Bangkok, Thailand; ^3^ Precision Pathology of Neoplasia Research Group Department of Pathology Faculty of Medicine Chulalongkorn University, Bangkok, Thailand; ^4^ Center of Excellence in Translational Hematology Faculty of Medicine Chulalongkorn University, Bangkok, Thailand

## Abstract

T-cell prolymphocytic leukemia (T-PLL) is a rare, mature T-cell leukemia which usually presents with aggressive behavior. We report an asymptomatic T-PLL patient diagnosed by clinical features, lymphocyte morphology, and flow cytometry. Incidentally, she was found to have lymphocytosis and lymphadenopathy. Flow cytometry from blood revealed an abnormally increased CD4+ T-cell population. T-cell receptor clonality assessment by next-generation sequencing revealed a dominant clone in the ß-chain constant region. No pathogenic mutations in 25 lymphoma-related genes were found. Due to her asymptomatic T-PLL disease, we observed her clinical situation and blood count every three months for at least one year.

## 1. Introduction

T-cell prolymphocytic leukemia (T-PLL) is a rare, mature T-cell neoplasm, accounting for 2% of mature lymphoid leukemia cases in adults [1]. The 2022 World Health Organization classification categorizes T-PLL as mature T-cell leukemia originating from clonal small to medium-sized post-thymic T prolymphocytes [2]. T-PLL was previously classified as T-cell chronic lymphocytic leukemia (CLL) and treated following the criteria of the International Workshop on Chronic Lymphocytic Leukemia (iwCLL) [3]. The clinical course and pathogenesis of T-PLL, however, are much different from CLL. The median age at diagnosis of T-PLL is approximately 65 years [1]. A common presentation is leukocytosis (white blood cells >100 × 10^9^/L) with anemia and thrombocytopenia [1]. Hepatosplenomegaly and generalized lymphadenopathy are also observed in patients with T-PLL [1]. The clinical course varies from asymptomatic disease to active disease. Asymptomatic T-PLL usually turns to a progressive symptomatic disease within 1-2 years [4]. Herein, we report a patient diagnosed with asymptomatic T-PLL, which, based on her previous blood tests, remained stable for more than four years.

## 2. Case Presentation

A 78-year-old Thai female patient was admitted due to andrographolide-induced acute kidney injury (creatinine of 4.86 mg/dL from the baseline of 0.7 mg/dL) from *Andrographis* herbal supplement use. Her medical history included hypothyroidism from Hashimoto's thyroiditis, diet-controlled dyslipidemia, and osteoarthritis of her right knee. Her complete blood count was 10.2 g/dL hemoglobin (Hb), 18.43 × 10^9^/L total white blood cells (WBC), which included an absolute lymphocyte count (ALC) at 14.38 × 10^9^/L, and normal platelet count (PLT) of 386 × 10^9^/L. She had a similar complete blood count documented four years prior (Hb at 11.4 g/dL, WBC at 12.99 × 10^9^/L, ALC at 8.55 × 10^9^/L, and PLT at 324 × 10^9^/L). She experienced no fever, drenching night sweats, significant weight loss, or lymphadenopathy. She could perform her basic and instrumental activities of daily living (Eastern Cooperative Oncology Group (ECOG) = 0) without problems. Her left supraclavicular lymph node was less than 1 cm. Her thyroid gland size was around 75 grams. Her spleen and liver size were within normal limits by physical examination. She had no abnormal skin rash. Her physical examination appeared normal for her cardiovascular and pulmonary systems. She denied any prior engagement in smoking, vaping, drinking, or using illicit drugs. She denied having any family history of cancer or abnormal chronic lymphocytosis. Her basic lab investigations, e.g., lactate dehydrogenase (LDH), uric acid, electrolytes, liver function test, blood urea nitrogen (BUN), and creatinine, were within normal limits. Her hepatitis B, hepatitis C, and human immunodeficiency virus (HIV) profiles were all negative.

**Figure 1:**
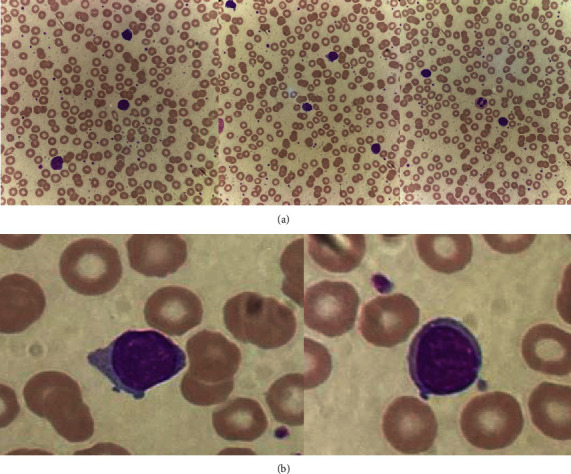
Peripheral blood smear showed increased small to medium-sized mature lymphocytes. (a) 100x; (b) 1,000x.

**Figure 2:**
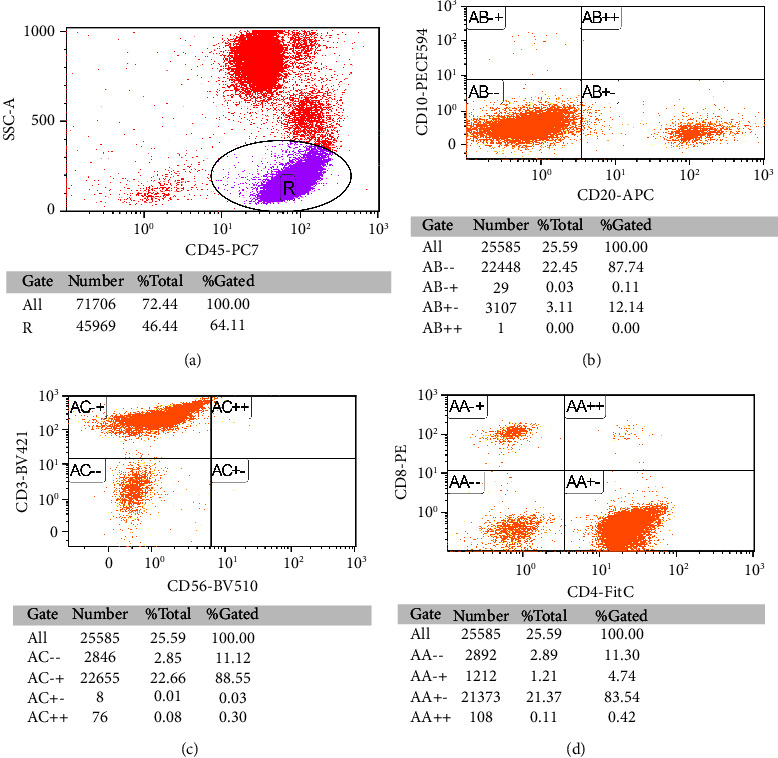
Flow cytometry from peripheral blood. (a) *R* represents lymphocyte gate and is further analyzed in (b)–(d).

**Figure 3:**
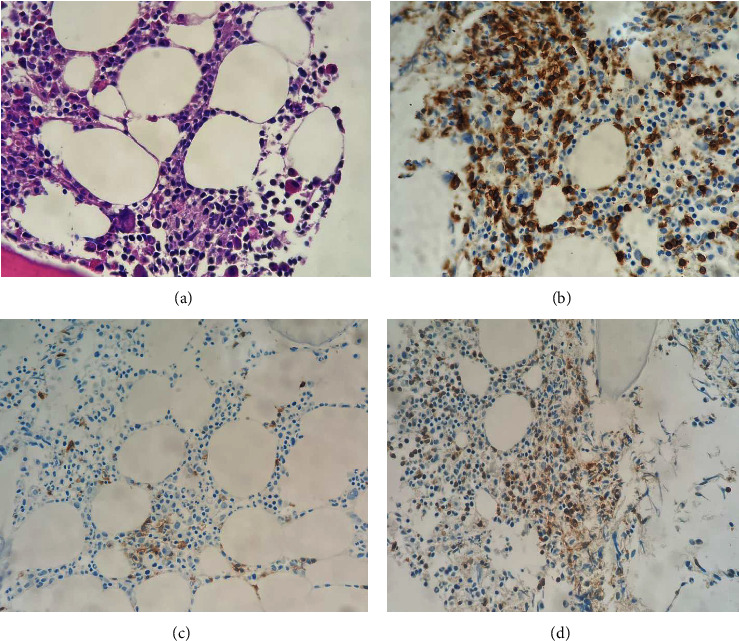
Immunohistochemistry of bone marrow biopsy showed CD3 and TCR-b positive with aberrant loss of CD7. (a) H&E (x400). (b) CD3 (x400). (c) CD7 (x400). (d) TCR-b (x400).

For additional investigations, her peripheral blood smear showed increased small mature lymphocytes with cytoplasmic blebs, as shown in Figure 1. Flow cytometry from her blood revealed an increased lymphocyte gate population (64%). The T-cell population (CD3+) accounted for 88% of total lymphocytes, while the B-cell population (CD20+) was detected in 12% of lymphocytes. Clonal B-cell could not be demonstrated by kappa or lambda restriction. A marked increase in the CD4+ T-cell population was shown with a CD4 : CD8 ratio of 84 : 5 (Figure 2). Bone marrow biopsy showed normocellular marrow containing 30–40 percent small to medium-sized atypical lymphoid cell proliferation. Immunohistochemistry revealed numerous CD3-positive cells with aberrant loss of CD7 (Figure 3). Combining the peripheral blood smear with the flow cytometry and bone marrow study, the results suggested a diagnosis of T-prolymphocytic leukemia. Her conventional chromosome analysis from bone marrow was normal (46, XX) for 20 metaphases.

**Figure 4:**
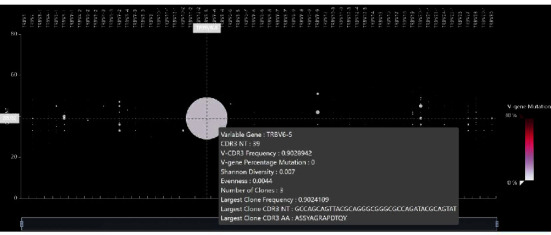
Results from next-generation sequencing showed a dominant clone of TRBV6-5 (90.28%).

DNA was extracted from her blood and then assessed for clonality using next-generation sequencing covering the T-cell receptor (TCR) beta chain constant region (Oncomine™ TCR Beta-SR Assay, Thermo Fisher Scientific) [5] using a 25-gene panel (Thermo Fisher Scientific) for detection of somatic mutations. Both panels were performed on the IonTorrent S5. The sequencing results revealed a dominant clone of TRBV6-5 (90.28%) (Figure 4). No pathogenic mutations were identified among the 25 lymphoma-related genes. The study was ethically approved by the Institutional Review Boards of Faculty of Medicine, Chulalongkorn University (IRB 776/66).

Due to her asymptomatic T-PLL, the treatment plan was to monitor her without intervention and follow up on her complete blood count and clinical features every three months.

## 3. Discussion

The latest 2022 World Health Organization classification of hematolymphoid tumors categorizes mature T-cell leukemia into four groups: T-prolymphocytic leukemia (T-PLL), T-large granular lymphocytic leukemia (T-LGLL), adult T-cell leukemia/lymphoma (ATLL), and Sezary syndrome [2]. The characterization of these mature T-cell leukemias is summarized in Table 1 [6–8]. T-PLL was previously classified as T-CLL, but the clinical manifestations and pathogenesis of T-PLL and CLL are very different [9]. T-cell prolymphocytic leukemia (T-PLL) is rare and occurs in about 2% of mature lymphoid leukemia cases in adults [1]. T-PLL is rare in East Asia, accounting for less than 1% of T-cell lymphoma [10–13]. In Thailand, from 2007 to 2020, there were no reports of T-PLL cases in our web-based registry system from 13 major Thai medical centers [14, 15]. To our knowledge, this report marks the first documented case of T-PLL in Thailand in nearly two decades.

T-PLL diagnosis requires three major criteria or two major criteria with one minor criterion [6]. Major criteria are >5 × 10^9^/L of T-PLL lymphocytes in peripheral blood or bone marrow, T-cell clonality, and abnormalities of 14q32 or Xq28 or expression of *TCL1A/B* or *MTCP1.* Minor criteria include abnormalities of chromosome 11 (11q22.3; *ATM*); abnormalities of chromosome 8 (idic(8), t(8; 8), trisomy 8q); abnormalities of chromosomes 5, 12, 13, and 22 or complex chromosome; and involvement of T-PLL specific site (splenomegaly or effusion) [6]. This patient fulfilled two major criteria (number of T-PLL lymphocytes and T-cell clonality). However, diagnosis should also be based on the morphology and immunophenotype of the lymphocytes from peripheral lymphocytes. Bone marrow study is usually necessary only for the pre- and post-treatment evaluation [6].

T-PLL lymphocytes are medium-sized with a high N/C ratio (nuclear/cytoplasmic), condensed chromatin with a nucleolus, and a clear basophilic blebbing cytoplasm. T-PLL lymphocytes could also have other forms, such as a small cell variant or a cerebriform variant. In addition to lymphocyte morphology, T-cell clonality should be investigated using next-generation sequencing (NGS) or polymerase chain reaction (PCR) for T-cell receptor genes (*TRB*, *TRG*) [6]. Negative serological testing or PCR testing for human T lymphotropic virus type 1 (HTLV1) is employed to differentiate from adult T-cell lymphocytic leukemia (ATLL). The immunophenotypes of post-thymic T-PLL cells are CD3+ (>80%), CD5+ (100%), CD7+ (>90%), CD4+ (60%), CD8+ (15%), and CD4+ 8+ (25%), together with the cytoplasmic expression of TCL1 (>90%) [6]. The lymphocyte counts of our patient with T-PLL exceeded 5 × 10^9^/L, demonstrating T-cell clonality of TRBV6-5. The immunophenotype from the flow cytometry of our patient showed that CD3-positive T cells predominate CD4 (84%) and are compatible with the T-PLL phenotype. Notably, our patient tested negative for CD7. However, it is worth mentioning that this phenotype has been reported in approximately 6% of T-PLL cases [4].

Rearrangements of T-cell leukemia/lymphoma 1 (TCL1) family genes, *TCL1A*, *MTCP1* (mature T-cell proliferation), and *TCL1B* (TCL1/MTCP1-like 1 (*TML1*)) are observed in more than 90% of T-PLL cases, including inv(14) (q11q32) or *t*(14; 14) (q11; q32) (involving *TCL1A* or *TCL1B*) or *t*(X; 14) (q28; q11) (involving MTCP1; mature T-cell proliferation) [6]. However, some cases did not have *TCL1* rearrangement but had classical clinical manifestation, morphology, and T-cell clonality compatible with T-PLL. These cases could be classified as *TCL1*-family negative T-PLL [6]. In our case, we did not observe any chromosomal abnormalities by conventional cytogenetic analysis. Fluorescence in situ hybridization [16] for specific locations, especially the *TCL1*-family gene, should be performed in suspicious cases.

For the indication for T-PLL treatment, only active disease should be treated, while asymptomatic T-PLL would be continuously observed. Criteria for active disease are constitutional symptoms (ECOG ≥ 2; unintentional weight loss of >10% in ≤6 months; drenching night sweats without infection; fever >38°C without infection), bone marrow failure (hemoglobin <10 g/dL; platelet <100 × 10^9^/L); enlarging lymph nodes >50% in 2 months or diameter doubling in less than six months, symptomatic enlarged lymph nodes, spleen, or liver, increasing lymphocytosis (>30 × 10^9^/L; >50% in 2 months; lymphocyte doubling time less than six months), and extranodal involvement (organ infiltration, peritoneal or pleural effusion, and central nervous system involvement) [17]. If any of these criteria were observed, treatment should be initiated. Among treatment options, anti-CD52 alemtuzumab has shown the best overall response rate of more than 90% and progression-free survival between 8 and 11 months. The recent recommendation for T-PLL is alemtuzumab induction treatment for 10–12 weeks in combination with allogeneic stem cell transplantation as consolidation therapy [17]. T-PLL usually presents with aggressive disease (70%). Our patient was asymptomatic, which showed no indication to start treatment.

## 4. Conclusion

In summary, T-PLL is a rare, mature T-cell lymphoma, especially in East Asia. T-PLL may present with lymphocytosis mimicking asymptomatic chronic lymphocytic leukemia. An appropriate diagnostic approach and investigations are helpful for the diagnosis of T-PLL.

## Figures and Tables

**Table 1:** Characteristics of mature T-cell leukemia include T-prolymphocytic leukemia (T-PLL), T-large granular lymphocytic leukemia (T-LGLL), and adult T-cell leukemia/lymphoma (ATLL).

Characteristics	T-PLL [6]	T-LGLL [7]	ATLL [8]
Clinical course	70% progressive 20–30% asymptomatic Lymphocytosis with hepatosplenomegaly and lymphadenopathy	Indolent Cytopenia Associated with autoimmune diseases	Associated with HTLV1 infection
Specific immunophenotype	TCL1+ CD7++	CD8+ CD56+ CD57+	CD4+ CD25+ CD7-
Lymphocyte morphology	Prolymphocytes with blebbing	Large granular lymphocytes	Flower cells
Cytogenetics	*t*(14; 14); inversion 14; *t*(X; 14)	*STAT*3, *STAT5b* mutation	Many
Treatment if indicated	Alemtuzumab Alem + pentostatin FMC AlloHSCT	CsA/pred MTX/pred Cyclophosphamide Fludarabine AlloHSCT	ZDV/IFN alpha CHO(M)P Mogamulizumab

Alem = alemtuzumab, FMC = fludarabine, mitoxantrone, and cyclophosphamide, AlloHSCT = allogeneic hematopoietic stem cell transplantation, CsA = cyclosporin A, Pred = prednisolone, MTX = methotrexate, ZDV = zidovudine, IFN = interferon, and CHO(M)P = cyclophosphamide, doxorubicin, vincristine, methotrexate, and prednisolone.

## Data Availability

Data are available on request due to privacy/ethical restrictions.
